# The psychosocial implication of childhood constipation on the children and family: A scoping review protocol.

**DOI:** 10.12688/hrbopenres.13713.1

**Published:** 2023-09-13

**Authors:** Yvonne McCague, Suja Somanadhan, Diarmuid Stokes, Eileen Furlong

**Affiliations:** 1 School of Nursing, Midwifery and Health Systems, University College Dublin, Dublin, Leinster, Ireland

**Keywords:** Children, constipation, psychosocial, implication, family, scoping review

## Abstract

**Background:** Constipation is a common problem in childhood that can have psychological, emotional, social, and health-related quality-of-life consequences on children and their families. Primary or functional constipation (FC) has no known underlying pathology but can be caused by lifestyle, psychological, and behavioural factors. Misdiagnosis and inadequate management of constipation can result in chronicity that can continue to adulthood, reducing quality of life for the child and their parents/family. It also causes emotional, psychological and emotional distress and concern for children and their families. This scoping review aims to answer the research question, “What has been reported about the psychosocial implication of childhood constipation among children and their families?”

**Methods:** The methodology for this scoping review will draw on the six stages of Arksey and O’Malley Framework and the updated and refined version by Levac *et al.* (2010) and Peters *et al.* (2022). The process and reporting will follow the PRISMA-ScR guidelines. The Population, Concept and Context (PCC) framework will guide the development of inclusion criteria and the search strategy for this scoping review. Systematic literature searches of PUBMED, CINAHL, ASSIA, PsycInfo and Cochrane Library will be conducted from inception to present. The critical appraisal will be performed on selected articles to promote trustworthiness and methodological rigour. Plans for consultation exercise and dissemination of findings will also be presented.

**Conclusion:** This scoping review aims to present a comprehensive synthesis of the characteristics and extent of available literature to develop an understanding of and identify gaps in current knowledge regarding the psychosocial implication of childhood constipation on children and their families.

## Introduction

Constipation is a defecation disorder that is an increasingly significant and prevalent chronic health problem in children ( Walter *et al.*, 2019). Despite this, it is often poorly recognised and inadequately treated ( Borowitz *et al.*, 2005; Widodo *et al*., 2018). For diagnosis purposes, constipation is categorised as primary or secondary based on its pathophysiology. Primary constipation, also known as functional constipation (FC), has no underlying organic causes and occurs in 95% of children diagnosed with constipation ( Koppen *et al.*, 2016; Tabbers *et al.*, 2014). In contrast, secondary constipation has organic pathology such as Hirschsprung’s disease, anorectal malformations, hypothyroidism, diabetes mellitus, hypercalcaemia and hypokalaemia ( Rajindrajith & Devanarayana, 2011). The diagnosis of primary or FC must exclude secondary constipation, as ‘red flag’ symptoms may point to a more serious underlying disorder requiring further investigations ( NICE, 2018).

While there is no known underlying cause, there are multiple factors that can lead to the development of constipation in children. Some environmental factors have been linked to a higher risk of this disorder in children for example: a sedentary lifestyle and poor diet, low socioeconomic background, poor maternal education, and cramped living accommodations ( Edan & Yahya, 2022; Kilincaslan *et al*., 2013; Ludvigsson *et al.*, 2006) as well as low levels of interaction with parents ( Yamada *et al*., 2019). Early childhood developmental factors have also been associated with constipation in young children, such as incidents of painful defecation at a young age leading to withholding behaviour (Kilincaslan *et al*., 2013; Mugie *et al.*, 2011); authoritarian parenting styles in younger age group (Kilincaslan *et al*., 2013; van Dijk *et al.*, 2015); stressful childhood incidents such as psychological trauma; physical, verbal and sexual abuse; being bullied in school and inadequate or punishment during toilet training ( Edan & Yahya, 2022; Inan *et al.*, 2007; Philips *et al.*, 2015; Rajindrajith *et al*., 2016; Rajindrajith *et al.*, 2021; Thomaz de Almeida *et al*., 2021; Walter *et al*., 2019) as well as alcoholism and severe illness within the family ( Oswari *et al*., 2018). 

Global epidemiological data on childhood constipation to date is limited. The available data on the prevalence of childhood constipation demonstrates a considerable variation between geographical regions ranging from 0.7% to 32% ( Chogle *et al.*, 2016; Játiva-Mariño, 2019; Oswari *et al*., 2018; Udoh *et al.*, 2017; van Tilburg *et al.*, 2015; Wu *et al*., 2011). In paediatric gastroenterology clinics, the reported prevalence ranges from 25% to 45% ( Benninga *et al.*, 2004; Di Lorenzo, 2000; Loening-Baucke, 2007). This variation may be attributed to a heterogeneity in definitions of constipation, duration of symptoms, methodology of studies, age distribution of children and epidemiological data ( Rajindrajith & Devanarayana, 2011). To date, there have been no epidemiological studies to identify the prevalence of childhood constipation in Ireland.

Childhood constipation has significant adverse consequences affecting multiple aspects of the life of children. Several studies have reported reduced average weight and height in children with chronic constipation when compared to children without the disorder ( Chang *et al.*, 2013; Pawłowska *et al.*, 2018; Yousefi *et al.*, 2019). Children with constipation also demonstrated higher somatic scores when compared to healthy children ( Appak *et al*., 2017; Kilincaslan *et al*., 2013; Rajindrajith *et al.*, 2013). Rajindrajith *et al.*’s (2013) cross-sectional survey using Pediatric Quality of Life Inventory and Child Somatization Index on children aged 13 to 18 reported higher somatisation scores with symptoms including headache, dizziness, lethargy, nausea and vomiting, as well as pain in the abdomen, lower back, chest and limbs/joints when compared to healthy children. This is supported by a prospective, comparative survey by Appak *et al.* (2017) on children with constipation between 4 and 18 years of age (n=32) and ‘healthy controls admitted to a paediatric gastroenterology outpatient clinic’ (n=31)(pg. 466). The study employed the following child and family assessment tools: Strengths and Difficulties Questionnaire (SDQ), Beck Depression Inventory (BDI), State and Trait Anxiety Inventory and Parental Attitude Research Instrument (PARI). High levels of somatic complaints including recurrent abdominal pain, frequency of micturition and faecal soiling were reported in this study. The study also reported significantly higher scores of anxiety and depression in children and their mothers, compared to healthy children and their mothers. This study has a few limitations which were not reported including small sample sizes and non-randomization of sampling which increases the risk of bias.

Chronic constipation also has a significant psychosocial impact on children and adolescents ( Appak *et al*., 2017; Kaugars *et al*., 2010; Kilincaslan *et al*., 2013; Rajindrajith *et al*., 2013; Rajindrajith *et al*., 2016; Rajindrajith *et al*., 2020; Ranasinghe *et al*., 2017; Wang *et al*., 2013). Wang *et al.*’s (2013) cross-sectional, case-control study on children aged 3 to 6 years of age using a Chinese version of the Pediatric Quality of Life Inventory ^TM^, version 4.0 (PEDQL ^TM^4.0) and Elkhayat *et al.*’s (2016) cross-sectional, case-control study on children aged 4 to 12 years of age using an Arabic versions of the Child Behavior Checklist for 4-18 years old (CBCL/4-18) and the Pediatric Quality of Life Inventory ^TM^, version 4.0 (PEDQL ^TM^4.0) found that scores for quality of life of children were lower compared to those of children without constipation. These studies also demonstrated that children with constipation had significantly higher anxiety levels, sleep disturbances and social problems. Sleep disturbance in children with constipation is also reported in Yildirim *et al*.’s (2017) quantitative study into health-related quality of life in school aged children. Kilincaslan *et al*.’s (2013) survey on children aged between 2 and 6 years (n=124) reported that children in the constipation group (n=65) demonstrated higher levels of psychological issues such as anxiety, depression, aggression, externalising and internalising compared to children with normal intestinal habits (n=59). This is supported by a more recent study by Rajindrajith *et al*.’s (2020), a cross-sectional survey which found that adolescents with constipation demonstrated increased risk of significant emotional, behavioural or internalising issues such as withdrawal, unresponsiveness, anxiety and depression in comparison to healthy adolescents.

Childhood constipation also has adverse psychosocial impact on the school environment of children and adolescents. Kaugars *et al*.’s (2010) qualitative study on children with chronic constipation (n=8) and their caregivers (n=8) found that 62.5% of children in the study reported that school performance was affected by constipation and faecal incontinence. The children in this study also expressed concerns of using toilets in the school and the negative effect of constipation and faecal incontinence at school. This is supported by Rajindrajith *et al*.’s (2016) survey using four validated instruments: ROME III diagnostic questionnaire for the paediatric functional GI disorders, child maltreatment questionnaire, Child Somatization Inventory and Pediatric Quality of Life Inventory (PedsQL). This study found that faecal incontinence associated with chronic constipation increased the likelihood of emotional abuse in school by other children and even teachers due to the faecal odour. This study emphasized the significance of the psychological impact of constipation and faecal incontinence on children and adolescents in terms of poor interaction, social phobia and academic performance.

Constipation with faecal incontinence has a greater adverse effect on the quality of life of the child when compared to childhood constipation without faecal incontinence ( Kovacic *et al*., 2015). This prospective study employed five validated instruments: PedsQL – Parent Report, PedsQL – Family Impact Module (FIM), Functional Disability Inventory (FDI) – Parent Version, Pediatric Inventory for Parents (PIP), and Pediatric Symptom checklist (PSC) – Parent Report. The study included families of children aged between 2 to 18 (n=410) found that quality of life scores worsened as the children’s ages increased. Consequently, adolescents with constipation and faecal incontinence demonstrated the lowest scores. Constipation with faecal incontinence also has a significant impact on families in terms of increased cost and time spent on laundry secondary to soiling and faecal incontinence ( Kaugars *et al*., 2010; Thompson *et al*., 2021a). There is also a significant cost impact of childhood constipation associated with utilisation of health care services such as emergency department and behavioural therapy, and prescriptions ( Kaugars *et al*., 2010; Liem *et al*., 2015; Thompson *et al*., 2021a).

Recurrent constipation as a result of treatment failures has a significant effect on the quality of life of the child’s parents and family ( Dolgun *et al*., 2013; Elkhayat *et al.*, 2016; Klages *et al*., 2017; Thompson *et al*., 2021b; Wang *et al.*, 2013), resulting in conflict within family members ( Kaugars *et al*., 2010). Parents and families of children with constipation have significantly lower quality of life scores in several aspects including physical, emotional, social, daily activity and relationship ( Wang *et al*., 2013). This is supported by Klages *et al*.’s (2017) study on caregivers of children aged between 2 and 18 using the Parental Opinion of Pediatric FC (POOPC) ( Silverman *et al*., 2015) which found that caregivers of children with constipation reported high levels of disease burden, distress and psychosocial problems. The study also found that caregivers were dissatisfied with management by healthcare providers which resulted in poor adherence to prescribed treatment. This supports findings from other studies including reports of parental anxiety, frustration, and mistrust of healthcare providers ( Kovacic *et al*., 2015) and Thompson *et al.'s* (2021b) qualitative study on parental experiences of caring for children with constipation which reported that parents expressed frustration and dissatisfaction with the care received in terms of dismissive and insensitive attitude of care providers, as well as inadequate care, support and information provided.

A review of current literature has highlighted that children with chronic constipation have high levels of behavioural problems such as withdrawal, anxiety, depression, aggression, externalising and internalising. Childhood constipation also has an adverse effect on the quality of life of children and their families, affecting their physical, emotional, social and school life, as well as daily activity and relationship. Parents have expressed anxiety and frustration, as well as dissatisfaction and distrust of healthcare professionals. To date, there has been no studies done to identify the psychosocial implication of childhood constipation on children and their families in Ireland.

The burden of chronic illness is one of the key focuses in the recently published Sláintecare Reports in Ireland, highlighting the need to reform the model of care of children and chronic conditions ( Houses of the Oireachtas, 2017). Therefore, addressing and identifying the impact of childhood constipation on children and their family is crucial to current needs and can be aligned with Sláintecare’s vision to improve child health and wellbeing services ( Houses of the Oireachtas, 2017). In addition, a recent study has highlighted constipation in children and young people as one of the highest-ranking priorities of research across the UK and Ireland ( Cathie *et al*., 2022).

A preliminary search of databases demonstrated an increasing interest in childhood constipation in recent years. The search was conducted through Cochrane Library on Systematic Reviews, Health Research Board (HRB) Open Research and Joanna Briggs Institute (JBI) Evidence Synthesis, as well as UCD Library Onesearch and Google Scholar where several reviews on constipation were identified. There were 6 reviews registered in PROSPERO: 30 systematic reviews and 2 scoping reviews in Google Scholar and one in HRB Open Research. The majority of all the reviews were focused on pharmacological and non-pharmacological treatments. There were three reviews identified with similar elements: Belsey *et al*.’s (2010) systematic review is focused on impact on quality of life in adults and children with constipation; Vriesman *et al*.’s (2019) systematic review is focused solely on the quality of life of children; Thompson *et al*.’s (2021a) systematic review is focused on experiences and information needs of parents. There are no recent reviews focused on the psychosocial implications of childhood constipation on children or the families of children with constipation. Childhood constipation is a complex health problem that has an adverse effect on multi-dimensional aspects of their life. Therefore, there is a need to identify the extent of these effects on the lives of children. In addition, the identification of how childhood constipation affects the wellbeing of the families is also crucial as the well-being of the child is dependent on family well-being ( Newland, 2015).

### Aims and objectives

This scoping review aims to identify and explore all current and available literature on the psychosocial implication of childhood constipation on the child and their family. Childhood constipation has a significant impact on multiple aspects of the child and their family’s lives. Therefore, this methodology’s broad approach will be appropriate and useful to map all available evidence from a heterogeneous source, clarify key concepts and identify gaps in the literature on the topic ( Tricco *et al.*, 2018). Scoping reviews are inherently flexible in its methodology allowing for the systematic and diverse search, including grey literature and Google scholar ( Peterson *et al.*, 2017:13). Therefore, it is suitable when exploring a complex and multi-dimensional concept such as constipation in children. The objective guiding this scoping review will be to:

1. Explore all available literature regarding the psychosocial implication of childhood constipation on children and families by identifying, critically appraising and synthesising relevant literature.
2. Describe the type and nature of available and current studies on the psychosocial implication of childhood constipation on the child and their families, the challenges they encounter, their views regarding support received; ways to improve psychosocial support.
3. Develop an understanding of the psychosocial implications that childhood constipation has on children and their families.
4. Guide the focus of subsequent research into this area.

## Methods

This scoping review will be guided by the six stages of Arksey and O’Malley Framework ( Arksey & O’Malley, 2005) and the updated and refined version by Levac *et al.* (2010) and Peters *et al.* (2022). In order to ensure rigour in reporting, the review will be carried out and reported using the Preferred Reporting Items for Systematic reviews and Meta-Analyses extension for Scoping Review (PRISMA-ScR) ( Tricco *et al.*, 2018). The six stages of the review are described as follows.

### Stage 1: Identifying the Research Question

The first stage in the Arksey and O’Malley’s Framework (2005) is the formulation of a research question that will address the key elements of the review and form the foundation of the search strategy. The Population, Concept and Context (PCC) framework is recommended for scoping reviews as it provides guidance in the formation of ‘clear and meaningful’ review questions and inclusion/exclusion criteria ( Peters *et al.*, 2022:2122). The review question should also be aligned to the review aim and purpose ( Westphaln *et al.*, 2021), with a broad scope to ensure extensive capture of relevant references ( Arksey & O’Malley, 2005). The review question formulated using the recommended PCC framework in consultation with research supervisors is as follows:

“What has been reported about the psychosocial implication of childhood constipation among children and their families?”

***Eligibility Criteria*.** The review question and objectives will determine eligibility criteria for this scoping review, as it will direct the development of inclusion and exclusion criteria ( Pollock *et al*., 2021). The PCC framework was employed to guide the development of the inclusion and exclusion criteria and the search strategy ( Peters *et al.*, 2022). All inclusion and exclusion criteria for this scoping review are described in Table 1, adapted from Boland, Cherry and Dickson’s (2017:82) screening and selection tool. Westphaln *et al*. (2021) has suggested that this stage requires a broad and iterative approach to refining the review questions, reviewing aim and objectives, and specifying elements of PCC.

**Table 1.** Inclusion and exclusion criteria of study selection.

**Review Question**	*What is the psychosocial impact of childhood constipation on children and their families?*	
	Inclusion	Exclusion
**Population**	Literature on human participants Children below 18 Families including biological and adoptive parents, (informal) caregivers that are part of the family unit, guardians, foster parents, siblings	Literature on non-human e.g. animals Adult above 18 Literature that does not include families of children with constipation
**Concept**	Literature that includes the impact of childhood constipation	Literature that does not include the impact of childhood constipation
**Context**	Literature that includes functional/idiopathic/ primary constipation in children below 18	Literature that does not include functional/idiopathic/primary constipation. Literature that includes constipation in adult above 18 Literature on secondary constipation Literature on other gastrointestinal disorder such as functional gastrointestinal disorder (FGID), slow transit constipation, infant dyschezia
**Study design**	Primary studies including quantitative, qualitative and mixed-methods	Case studies or reports, expert opinions, editorials and literature reviews
**Geographical** **location**	No limitation	
**Period**	Literature published from 2000 to current	Literature published prior to 2000
**Language**	English language articles	Non-English articles

***Population*:** This scoping review will focus on the psychosocial implication of childhood constipation among children and their families. Therefore, the review will include studies that consider the psychosocial implication from the perspective of children (aged below 18 years living with constipation), and their families including biological and adoptive parents; siblings; formal and informal caregivers; guardians and foster parents that form part of the family unit.

***Concept*:** The concept of interest is the psychosocial implication of childhood constipation on children and their families. This includes the direct and indirect psychosocial impacts of childhood constipation on children and their family members. How these impacts are measured (e.g. using validated screening or measurement tools) and experience studies will also be mapped.

***Context*:** The context of this scoping review is childhood constipation. The review will be limited to children under the age of 18 years experiencing primary or functional constipation.

No limitation will be placed on geographical locations as the objective is to explore all available literature regarding the psychosocial impact of childhood constipation. The search will be limited to English language articles as this is the primary language of researchers and due to time, cost and translation constraints. There have been reports regarding the potential of excluding vital evidence and language bias when limiting reviews to English-language studies ( Boland *et al*., 2017). However, the evidence is inconclusive as some studies have demonstrated limited evidence of bias when limiting language in systematic reviews and meta-analysis ( Dobrescu *et al.,* 2021; Morrison *et al*., 2012). In cognisant of this, the limitation to English language articles will be reported in the scoping review.

Studies for inclusion in this scoping review are primary studies including quantitative, qualitative and mixed-methods/multi-methods that explore the psychosocial implications of childhood constipation on children and their families. At the first stage of identifying relevant studies, abstract and full-text will be considered in order to ensure a wide capture of all available relevant studies ( Peters *et al.*, 2022). Literature that is not primary studies such as case studies or reports, expert opinions, editorials and literature reviews will be excluded.

### Stage 2: Identifying Relevant Studies

Constipation has multifactorial contributing factors including lifestyle, child development issues, and psychological and behavioural disorders. It also has an adverse effect on multi-dimensional aspects of the life of the child and their family. Therefore, a comprehensive search of electronic databases will include literature from allied healthcare disciplines including PUBMED, CINAHL, ASSIA, Psycinfo and Cochrane Library. Grey literature including ‘OpenGray’ and Google Scholar will also be searched to ensure a comprehensive search. The literature will also be supplemented by hand searching reference lists of included articles.

The university librarian (DS) was consulted to ensure the development of an extensive and comprehensive search strategy. The role of librarians in the development of systematic and scoping reviews is increasingly recognized as being invaluable. It is recommended that librarians are consulted at early stages especially at stage 1 and 2 of scoping reviews to maximise rigour and validity of the review ( Morris *et al*., 2016). The university librarian’s (DS) consultation commenced with the identification of key words and index terms of the major elements within the PCC framework - childhood constipation, children, families, and psychosocial implication. All possible synonymous words and matching index terms using controlled vocabulary were identified using synonym and thesaurus software tool as well as MeSH tool in PUBMED and Subject Headings in CINAHL to ensure all relevant articles will be included ( Bramer *et al*., 2018). To further broaden the search, truncation was used. Once syntax appropriate to each database including parentheses was refined, Boolean operators and field codes were employed ( Bramer *et al*., 2018). Boolean search strings by keywords and subject headings are detailed in *Extended data* ( McCague, 2023).

Once keywords and subject headings are refined, the search strategy can commence and will be guided by the JBI three-step search strategy Peters *et al.*’s (2022).

1. The first stage involves a preliminary limited search of two databases, PUBMED and CINAHL to identify relevant articles. A full search strategy will be developed from key words identified from titles and abstract, as well as index terms used in the relevant articles. An example of search strategy using PUBMED is available in *Extended data* ( McCague, 2023).
2. The second stage will consist of a search using all identified key words and index terms in the remaining selected databases.
3. Hand searching through reference lists of all selected articles will be performed at this stage. The search strategy stage for scoping reviews is also an iterative process as additional keywords and search terms can be identified during the search process, which will be incorporated into the search strategy ( Peters *et al.*, 2022). The complete search strategy and results will be documented and described in detail, including any alterations to demonstrate transparency. The librarian (DS) will again be consulted during this stage to ensure a comprehensive and thorough search.

### Stage 3: Study Selection

The JBI Reviewers Manual for Scoping Reviews will be employed to guide the study selection process. EndNote 20 will be used to manage bibliographic references where all pertinent literature identified will be saved and organized to enable easy retrieval and generation of the reference list. Covidence will then be used to further manage results of the search by importing and screening articles, extracting data and removal of duplicates ( Covidence, 2022).

The process will involve:

- 1 ^st^ (YM) and 2 ^nd^ review author will pre-screen all results from the search strategy independently to identify eligible studies based on title and abstract.
- Pilot testing of random selection of 25 titles and abstracts of selected articles will be performed using Covidence to confirm consistency ( Peters *et al.*, 2022).
- Potentially eligible studies that meet all inclusion criteria will undergo detailed full-text screening by the 1 ^st^ review author (YM). Uncertainty of articles for inclusion will be screened independently by 2nd review author.
- The 3rd review author will resolve any eligibility disagreements by independently screening selected articles (SS/EF).

The selection process will be described in detail, including the number of articles identified, screened for eligibility, and included and excluded to ensure transparency and replicability ( Tricco *et al*., 2018). Description will be in narrative format as well as presented in a PRISMA flow diagram such as one in *Extended Data* ( McCague, 2023). Justifications will be provided for articles that are excluded.

### Stage 4: Charting the Data

The data charting process will be undertaken using a recognized tool such as JBI Template Data Extraction Instrument ( Peters *et al.*, 2022) to ensure transparency, consistency and reduce bias (see *Extended Data,* McCague, 2023). This tool will be adapted to meet this scoping review’s question and objectives. It will be piloted on a minimum of two randomly selected included articles by two reviewers independently as recommended to ensure consistency ( Pollock *et al.*, 2021). Any incongruity in the data charted will result in a revision of the data charting form. All alterations made to the original tool will be documented and described to ensure transparency in reporting. Key information to be collected will include (but not restricted to) author, year of publication, country of origin, study design and methodology, aims and purpose of study, population and sample size, outcomes and key findings of study. The information collated will be charted in a data charting form using Microsoft Excel following data extraction tool templates from JBI ( Peters *et al.*, 2022).

Critical appraisal and assessment of selected studies are not prerequisites in scoping reviews ( Pollock *et al.*, 2021). However, studies should be systematically and critically appraised to promote trustworthiness and methodological rigour ( Levac *et al.*, 2010; Tod *et al*., 2022). For this purpose, many appraisal tools are specifically designed for various types of studies. These include the JBI critical appraisal tools ( Joanna Briggs Institute, 2016), the Critical Appraisal Skills Programme (CASP) ( Critical Appraisal Skills Programme, 2022), the Mixed Method Appraisal Tool (MMAT) ( Hong *et al*., 2018) and the Appraisal of Guidelines for Research and Evaluation II (Agree II) ( Brouwers *et al*., 2010). 

### Stage 5: Collating, Summarizing and Reporting the Results

Results of the search and screening process will be demonstrated in a PRISMA-ScR flowchart ( Tricco *et al.*, 2018). Data will be mapped and presented using the PCC inclusion criteria elements in a tabular presentation ( Peters *et al.*, 2022). As data in scoping reviews will be predominantly descriptive, data will be synthesised quantitative and qualitatively. Data such as year and country of publication, population and sample size will be analysed using SPSS Version 24 and described using descriptive statistics. Data will be presented through narrative description as well as visual representations such as tables, pie charts and bubble plots ( Pollock *et al.*, 2021) rather than synthesized format as the aim is to present and describe the ‘breadth of the literature and identify priority areas’ ( Westphaln *et al.*, 2021:10). This scoping review will be guided by Levac *et al.*’s (2010) three-step process:

1. Perform quantitative descriptive analysis and qualitative thematic analysis.
2. Frame results in the context of the research question, aim and PCC.
3. Frame findings in the context of future studies.

### Stage 6: Consultation Exercise and Dissemination

Consultation with key stakeholders is an optional but valuable stage as it is an opportunity to ensure the search strategy adequately includes all appropriate terms as well as to obtain feedback on findings ( Roberts *et al.*, 2021). While the involvement of knowledge users is not required at every stage, consultation at early stages can be beneficial (Pollock *et al.*, 2022). Key stakeholders to be consulted will include experts in paediatric constipation, emergency medicine paediatricians, paediatric gastroenterologists, general paediatricians, Advanced Nurse Practitioners and Clinical Nurse Specialists in Paediatric Emergency Departments and Gastroenterology Clinics, General Practitioners (GP), continence nurses and dieticians. Stakeholders within other allied healthcare disciplines such as sociology, psychology, and behavioural science with an interest in childhood constipation will also be consulted. The Children’s Research Network (CRN) of Ireland and Northern Ireland will also be consulted, specifically the Early Childhood Research Group (ECRG) whose aim is to support, connect and inform the early childhood research community. Other stakeholders may include members of the public through Children’s Health Ireland Family Forum and Youth Advisory Council.

Findings from the scoping review will be disseminated using various strategies including presentations at national and international conferences as well as through publications in peer-reviewed journals. Findings will also be shared with appropriate groups such as the ECRG at CRN which has members that encompass a wide variety of individuals including academics and non-academics in the community, voluntary and social enterprise bodies, and government and statutory bodies. This will assist in a wide dissemination of the scoping review findings. In addition, findings from the review will be presented to appropriate key stakeholders and HSCPs involved in the management of the paediatric population in acute and non-acute settings nationally.

## Study Status

At the time of publication, this study is at the stage of preliminary searches of databases.

## Discussion

There is a growing recognition that childhood constipation is an increasing global health problem which results in significant burden on the child and their family, health services, society and economy. Studies to date have identified that childhood constipation has a significant implication on health-related quality of life on the children and their families, affecting multiple domains of children and their families’ lives including physical, emotional, school and psychosocial, as well as daily activity and relationship.

To date, no studies have identified the psychosocial implication of childhood constipation on children and their families in Ireland. Therefore, there is a need for a systematic scoping review to identify and explore all available evidence regarding the psychosocial implication of childhood constipation on the children and their families, as well as to identify gaps in the literature. Findings from the review will be used to direct the focus of subsequent research into childhood constipation in Ireland.

This protocol has been developed to guide a scoping review into the impact of childhood constipation on children and their families. Scoping reviews are inclined to be iterative especially in the search strategy, data extraction and data presentation stages, which can often result in deviance from the protocol ( Peters *et al.*, 2022). This is acceptable and recommended to promote the development of a thorough, systematic and comprehensive review. To ensure transparency and rigour, all amendments will be clearly documented and described.

### Ethics statement

Ethical approval will not be required as the scoping review outlined in this protocol will review publicly available literature. 

## Data Availability

There is no underlying data associated with this article. Open Science Framework: ‘The psychosocial implication of childhood constipation on the children and family: A scoping review protocol.’ https://doi.org/10.17605/OSF.IO/EQDBJ. This project contains the following extended data: Boolean search strings by keywords and subject headings PUBMED and Boolean search strings by keywords and subject headings CINAHL Search Strategy PUBMED PRISMA flow diagram for study selection process Data Charting from Sample Extended data are available under the terms of the Creative Commons Zero "No rights reserved" data waiver (CC0 1.0 Public domain dedication).

## References

[ref-2] Appak YÇ Sapmaz ŞY Doğan G : Clinical findings, child and mother psychosocial status in functional constipation. *Turk J Gastroenterol.* 2017;28(6):465–70. 10.5152/tjg.2017.17216 29086714

[ref-3] Arksey H O’Malley L : Scoping studies: towards a methodological framework. *Int J Soc Res Methodol.* 2005;8(1):19–32. 10.1080/1364557032000119616

[ref-101] Belsey J Greenfield S Candy D : Systematic review: impact of constipation on quality of life in adults and children. *Alimentary Pharmacology & Therapeutics.* 2010;31(9):938–949. 10.1111/j.1365-2036.2010.04273.x 20180788

[ref-6] Benninga MA Voskuijl WP Taminiau JAJM : Childhood constipation: is there new light in the tunnel? *J Pediatr Gastroenterol Nutr.* 2004;39(5):448–64. 10.1097/00005176-200411000-00002 15572881

[ref-7] Boland A Cherry G Dickson R : Doing a Systematic Review: A Student’s Guide.2 ^nd^Ed. London: Sage,2017. Reference Source

[ref-9] Borowitz SM Cox DJ Kovatchev B : Treatment of childhood constipation by primary care physicians: efficacy and predictors of outcome. *Pediatrics.* 2005;115(4):873–877. 10.1542/peds.2004-0537 15805358

[ref-10] Bramer WM de Jonge GB Rethlefsen ML : A systematic approach to searching: an efficient and complete method to develop literature searches. *J Med Libr Assoc.* 2018;106(4):531–541. 10.5195/jmla.2018.283 30271302 PMC6148622

[ref-11] Brouwers MC Kho ME Browman GP : AGREE II: advancing guideline development, reporting and evaluation in health care. *J Clin Epidemiol.* 2010;63(12):1308–1311. 10.1016/j.jclinepi.2010.07.001 20656455

[ref-83] Cathie K Sutcliffe AG Bandi S : Priorities for child health research across the UK and Ireland. *Arch Dis Child.* 2022;107(5):474–478. 10.1136/archdischild-2021-322636 34716174

[ref-13] Chang SH Park KY Kang SK : Prevalence, clinical characteristics, and management of functional constipation at pediatric gastroenterology clinics. *J Korean Med Sci.* 2013;28(9):1356–1361. 10.3346/jkms.2013.28.9.1356 24015043 PMC3763112

[ref-14] Chogle A Velasco-Benitez CA Koppen IJ : A Population-Based Study on the Epidemiology of Functional Gastrointestinal Disorders in Young Children. *J Pediatr.* 2016;179:139–143.e1. 10.1016/j.jpeds.2016.08.095 27726867

[ref-18] Covidence: Covidence Systematic Review Software.Veritas Health Innovation, Melbourne, Australia,2022. Reference Source

[ref-19] Critical Appraisal Skills Programme: Critical Appraisal Skills Programme.2022; Accessed 25/02/2023. Reference Source

[ref-102] Di Lorenzo C : Childhood constipation: finally some hard data about hard stools! *J Pediatr.* 2000;136(1):4–7. 10.1016/s0022-3476(00)90039-8 10636964

[ref-86] Dobrescu AI Nussbaumer-Streit B Klerings I : Restricting evidence syntheses of interventions to English-language publications is a viable methodological shortcut for most medical topics: a systematic review. *J Clin Epidemiol.* 2021;137:209–217. 10.1016/j.jclinepi.2021.04.012 33933579

[ref-87] Dolgun E Yavuz M Celik A : The effects of constipation on the quality of life of children and mothers. *Turk J Pediatr.* 2013;55(2):180–185. 24192678

[ref-22] Edan OAA Yahya FS : Socio-demographic characteristics and risk factors of functional constipation in children: A case control study. *Sri Lanka J Child Health.* 2022;51(2):227–234. 10.4038/sljch.v51i2.10123

[ref-23] Elkhayat HA Shehata MH Nada A : Impact of functional constipation on psychosocial functioning and quality of life of children: a cross sectional study. *Egypt Paediatr Assoc Gaz.* 2016;64(3):136–141. 10.1016/j.epag.2016.05.003

[ref-24] Hong QN Gonzalez-Reyes A Pluye P : Improving the usefulness of a tool for appraising the quality of qualitative, quantitative and mixed methods studies, the Mixed Methods Appraisal Tool (MMAT). *J Eval Clin Pract.* 2018;24(3):459–467. 10.1111/jep.12884 29464873

[ref-91] Houses of the Oireachtas: Committee on the Future of Healthcare: Slaintecare Report. Houses of the Oireachtas, Dublin,2017. Reference Source

[ref-103] Inan M Aydiner CY Tokuc B : Factors associated with childhood constipation. *J Paediatr Child Health.* 2007;43(10):700–706. 10.1111/j.1440-1754.2007.01165.x 17640287

[ref-27] Játiva-Mariño E Rivera-Valenzuela MG Velasco-Benitez CA : The prevalence of functional constipation in children was unchanged after the Rome IV criteria halved the diagnosis period in Rome III. *Acta Paediatr.* 2019;108(12):2274–2277. 10.1111/apa.14880 31140192

[ref-28] Joanna Briggs Institute (JBI): Critical Appraisal Tools.2016; Accessed 25/02/2023. Reference Source

[ref-92] Kaçer EÖ Kaçer İ : The burden of pediatric emergency departments, constipation: a systematic review. *Egypt Pediatric Association Gaz.* 2022;70(1):1–6. 10.1186/s43054-022-00101-6

[ref-93] Kaugars A Silverman A Kinservik M : Families' perspectives on the effect of constipation and fecal incontinence on quality of life. *J Pediatr Gastroenterol Nutr.* 2010;51(6):747–752. 10.1097/MPG.0b013e3181de0651 20706148

[ref-104] Kilincaslan H Abali O Demirkaya SK : Clinical, psychological and maternal characteristics in early functional constipation. *Pediatr Int.* 2014;56(4):588–593. 10.1111/ped.12282 24373103

[ref-105] Klages KL Berlin KS Silverman AH : Empirically Derived Patterns of Pain, Stooling, and Incontinence and Their Relations to Health-Related Quality of Life Among Youth With Chronic Constipation. *J Pediatr Psychol.* 2017;42(3):325–334. 10.1093/jpepsy/jsw068 27474732

[ref-31] Koppen IJN Kuizenga-Wessel S Lu PL : Surgical decision-making in the management of children with intractable functional constipation: What are we doing and are we doing it right? *J Pediatr Surg.* 2016;51(10):1607–1612. 10.1016/j.jpedsurg.2016.05.023 27329390

[ref-107] Kovacic K Sood MR Mugie S : A Multicenter Study on Childhood Constipation and Fecal Incontinence: Effects on Quality of Life. *J Pediatr.* 2015;166(6):1482–1487. e1481. 10.1016/j.jpeds.2015.03.016 26008173

[ref-108] Levac D Colquhoun H O'Brien KK : Scoping studies: advancing the methodology. *Implement Sci.* 2010;5(1):69. 10.1186/1748-5908-5-69 20854677 PMC2954944

[ref-32] Liem O Harman J Benninga M : Health Utilization and Cost Impact of Childhood Constipation in the United States. *J Pediatr.* 2009;154(2):258–262. 10.1016/j.jpeds.2008.07.060 18822430

[ref-106] Loening-Baucke V : Prevalence rates for constipation and faecal and urinary incontinence. *Arch Dis Child.* 2007;92(6):486–489. 10.1136/adc.2006.098335 16857698 PMC2066162

[ref-35] Ludvigsson JF Abis Study Group : Epidemiological study of constipation and other gastrointestinal symptoms in 8000 children.Gastrointestinal symptoms in children. *Acta Paediatr.* 2006;95(5):573–580. 10.1111/j.1651-2227.2006.tb02286.x 16825138

[ref-109] McCague Y : Extended Data for ‘The psychosocial implication of childhood constipation on the children and family: A scoping review protocol.’ 2023. 10.17605/OSF.IO/EQDBJ PMC11134151 38812827

[ref-39] Morris M Boruff JT Gore GC : Scoping reviews: establishing the role of the librarian. *J Med Libr Assoc.* 2016;104(4):346–354. 10.3163/1536-5050.104.4.020 27822163 PMC5079503

[ref-40] Morrison A Polisena J Husereau D : The effect of English-language restriction on systematic review-based meta-analyses: a systematic review of empirical studies. *Int J Technol Assess Health Care.* 2012;28(2):138–144. 10.1017/S0266462312000086 22559755

[ref-41] Mugie SM Di Lorenzo C Benninga MA : Constipation in childhood. *Nat Rev Gastroenterol Hepatol.* 2011;8(9):502–511. 10.1038/nrgastro.2011.130 21808283

[ref-42] National Institute for Health and Care Excellence (NICE): 2018 Exceptional Surveillance of Constipation in Children: Diagnosis and Management (NICE Guideline CG99).2018; Accessed 01/01/2023. 31846251

[ref-111] Newland LA : Family well-being, parenting, and child well‐being: Pathways to healthy adjustment. *Clinical Psychologist.* 2015;19(1):3–14. 10.1111/cp.12059

[ref-110] Oswari H Alatas FS Hegar B : Epidemiology of Paediatric constipation in Indonesia and its association with exposure to stressful life events. *BMC Gastroenterol.* 2018;18(1): 146. 10.1186/s12876-018-0873-0 30285647 PMC6171310

[ref-45] Pawłowska K Umławska W Iwańczak B : A link between nutritional and growth states in pediatric patients with functional gastrointestinal disorders. *J Pediatr.* 2018;199:171–177. 10.1016/j.jpeds.2018.02.069 29709346

[ref-46] Peters MDJ Godfrey C McInerney P : Best practice guidance and reporting items for the development of scoping review protocols. *JBI Evid Synth.* 2022;20(4):953–968. 10.11124/JBIES-21-00242 35102103

[ref-47] Peterson J Pearce PF Ferguson LA : Understanding scoping reviews: definition, purpose, and process. *J Am Assoc Nurse Pract.* 2017;29(1):12–16. 10.1002/2327-6924.12380 27245885

[ref-48] Philips EM Peeters B Teeuw AH : Stressful Life Events in Children With Functional Defecation Disorders. *J Pediatr Gastroenterol Nutr.* 2015;61(4):384–392. 10.1097/MPG.0000000000000882 26192701

[ref-49] Pollock D Davies EL Peters MDJ : Undertaking a scoping review: A practical guide for nursing and midwifery students, clinicians, researchers, and academics. *J Adv Nurs.* 2021;77(4): 2102–2113. 10.1111/jan.14743 33543511 PMC8049063

[ref-52] Rajindrajith S Devanarayana NM : Constipation in children: novel insight into epidemiology, pathophysiology and management. *J Neurogastroenterol Motil.* 2011;17(1):35–47. 10.5056/jnm.2011.17.1.35 21369490 PMC3042216

[ref-112] Rajindrajith S Devanarayana NM Benninga MA : Fecal Incontinence in Adolescents Is Associated With Child Abuse, Somatization, and Poor Health-related Quality of Life. *J Pediatr Gastroenterol Nutr.* 2016;62(5):698–703. 10.1097/MPG.0000000000001006 26485604

[ref-114] Rajindrajith S Devanarayana NM Thapar N : Functional Fecal Incontinence in Children: Epidemiology, Pathophysiology, Evaluation, and Management. *J Pediatr Gastroenterol Nutr.* 2021;72(6):794–801. 10.1097/MPG.0000000000003056 33534361

[ref-53] Rajindrajith S Devanarayana NM Weerasooriya L : Quality of life and somatic symptoms in children with constipation: a school-based study. *J Pediatr.* 2013;163(4):1069–72. e1. 10.1016/j.jpeds.2013.05.012 23800401

[ref-113] Rajindrajith S Ranathunga N Jayawickrama N : Behavioral and emotional problems in adolescents with constipation and their association with quality of life. *PLoS One.* 2020;15(10): e0239092. 10.1371/journal.pone.0239092 33044960 PMC7549826

[ref-115] Ranasinghe N Devanarayana NM Benninga MA : Psychological maladjustment and quality of life in adolescents with constipation. *Arch Dis Child.* 2017;102(3):268–273. 10.1136/archdischild-2016-310694 27402734

[ref-54] Roberts K Casey M Coghlan D : A scoping review protocol to map the evidence on the use of action research methodology by healthcare professionals and in healthcare team settings [version 1; peer review: 1 approved]. *HRB Open Res.* 2021;4:68. 10.12688/hrbopenres.13275.1 38800821 PMC11116937

[ref-116] Silverman AH Berlin KS Di Lorenzo C : Measuring Health-Related Quality of Life With the Parental Opinions of Pediatric Constipation Questionnaire. *J Pediatr Psychol.* 2015;40(8):814–824. 10.1093/jpepsy/jsv028 25840448

[ref-58] Tabbers MM DiLorenzo C Berger MY : Evaluation and treatment of functional constipation in infants and children: evidence-based recommendations from ESPGHAN and NASPGHAN. *J Pediatr Gastroenterol Nutr.* 2014;58(2):258–274. 10.1097/MPG.0000000000000266 24345831

[ref-117] Thomaz de Almeida CN Tahan S Areco KN : Association between abuse and neglect with functional constipation and irritable bowel syndrome in adolescents. *Scand J Gastroenterol.* 2021;56(10):1146–1151. 10.1080/00365521.2021.1923059 34469265

[ref-119] Thompson AP MacDonald SE Wine E : Understanding Parents' Experiences When Caring for a Child With Functional Constipation: Interpretive Description Study. *JMIR Pediatr Parent.* 2021b;4(1): e24851. 10.2196/24851 33470939 PMC7857943

[ref-118] Thompson AP Wine E MacDonald SE : Parents’ Experiences and Information Needs While Caring for a Child With Functional Constipation: A Systematic Review. *Clin Pediatr (Phila).* 2021a;60(3):154–169. 10.1177/0009922820964457 33026251

[ref-120] Tod D Booth A Smith B : Critical appraisal. *Int Rev Sport Exerc Psychol.* 2022;15(1):52–72. 10.1080/1750984X.2021.1952471

[ref-62] Tricco AC Lillie E Zarin W : PRISMA Extension for Scoping Reviews (PRISMA-ScR): Checklist and Explanation. *Ann Intern Med.* 2018;169(7):467–473. 10.7326/M18-0850 30178033

[ref-64] Udoh EE Rajindrith S Devanarayana NM : Prevalence and risk factors for functional constipation in adolescent Nigerians. *Arch Dis Child.* 2017;102(9):841–844. 10.1136/archdischild-2016-311908 28446425

[ref-65] van Dijk M de Vries GJ Last BF : Parental child-rearing attitudes are associated with functional constipation in childhood. *Arch Dis Child.* 2015;100(4):329–333. 10.1136/archdischild-2014-305941 25359759

[ref-66] van Tilburg MA Hyman PE Walker L : Prevalence of functional gastrointestinal disorders in infants and toddlers. *J Pediatr.* 2015;166(3):684–9. 10.1016/j.jpeds.2014.11.039 25557967

[ref-68] Walter AW Hovenkamp A Devanarayana NM : Functional constipation in infancy and early childhood: epidemiology, risk factors, and healthcare consultation. *BMC Pediatr.* 2019;19(1): 285. 10.1186/s12887-019-1652-y 31416431 PMC6694472

[ref-69] Wang C Shang L Zhang Y : Impact of functional constipation on health-related quality of life in preschool children and their families in Xi'an, China. *PLoS One.* 2013;8(10): e77273. 10.1371/journal.pone.0077273 24130872 PMC3795078

[ref-70] Westphaln KK Regoeczi W Masotya M : From Arksey and O'Malley and Beyond: Customizations to enhance a team-based, mixed approach to scoping review methodology. *MethodsX.* 2021;8: 101375. 10.1016/j.mex.2021.101375 34430271 PMC8374523

[ref-72] Widodo A Hegar B Vandenplas Y : Pediatricians lack knowledge for the diagnosis and management of functional constipation in children over 6 mo of age. *World J Clin Pediatr.* 2018;7(1):56–61. 10.5409/wjcp.v7.i1.56 29456933 PMC5803566

[ref-121] Wu TC Chen LK Pan WH : Constipation in Taiwan elementary school students: a nationwide survey. *J Chin Med Assoc.* 2011;74(2):57–61. 10.1016/j.jcma.2011.01.012 21354081

[ref-122] Yamada M Sekine M Tatsuse T : Psychological Stress, Family Environment, and Constipation in Japanese Children: The Toyama Birth Cohort Study. *J Epidemiol.* 2019;29(6):220–226. 10.2188/jea.JE20180016 30146529 PMC6522390

[ref-123] Yıldırım Ş Kaymaz N Tekin M : Health Related Quality of Life and the Quality of Sleep in School Aged Children with Functional Constipation. *Compr Child Adolesc Nurs.* 2017;40(1):53–61. 10.1080/24694193.2016.1273976

[ref-74] Yousefi A Mohamadian S Sharifabadi P : How does functional constipation affect growth status in children? *Iranian J Pediatr.* 2019;29(2): e85700. 10.5812/ijp.85700

