# Supplementary material for: The psychosocial implication of childhood constipation on the children and family: A scoping review protocol
Source: HRB Open Res. 2024 Jun 18;6:48. Originally published 2023 Sep 13. [Version 2] doi: 10.12688/hrbopenres.13713.2 (PMC11134151; doi:10.12688/hrbopenres.13713.2)
Supplement: Peer review history

# Reviewer report: Reviewer response for version 2

**DOI:** 10.21956/hrbopenres.15258.r40807

**Published:** 2024-06-27

**Authors:** Carol Joinson

**Affiliations:** 1 Centre for Academic Child Health, Population Health Sciences, Bristol Medical School, University of Bristol, Bristol, England, UK

The authors' revisions to the protocol are satisfactory.

Is the study design appropriate for the research question?

Yes

Is the rationale for, and objectives of, the study clearly described?

Yes

Are sufficient details of the methods provided to allow replication by others?

Yes

Are the datasets clearly presented in a useable and accessible format?

Not applicable

Reviewer Expertise:

Paediatric incontinence; developmental psychology; epidemiology; mental health

I confirm that I have read this submission and believe that I have an appropriate level of expertise to confirm that it is of an acceptable scientific standard.

# Reviewer report: Reviewer response for version 1

**DOI:** 10.21956/hrbopenres.14999.r37444

**Published:** 2024-05-28

**Authors:** Nilton Machado

**Affiliations:** 1 Pediatrics, São Paulo State University, Botucatu, SP, Brazil; 2 Division of Pediatric Gastroenterology, Hepatology, and Nutrition, Department of Pediatrics, Botucatu Medical School, São Paulo State University, Sao Paulo, Brazil

The psychosocial implication of childhood constipation on the children and family: A scoping review protocol.

 To identify and explore all current and available literature on the psychosocial implication of childhood constipation on the child and their family, the authors developed this study protocol, titled” ****The psychosocial implication of childhood constipation on the children and family: A scoping review protocol.” This study is of great importance, as there is currently a tendency to include the approach of psychosocial problems in the initial management of children and adolescents with Constipation.

**Abstract**. Clearly described

**Introduction**. The authors, with an adequate literature citation, address a wide range of problems related to Constipation in children and adolescents, such as environmental factors, stressful life events, abuse, bullying, punishment, interaction with parents, alcoholism, illness within the family, anxiety and depression, sleep disturbance, painful defecation, withholding behavior. These topics are fully experienced in a clinic that treats functional gastrointestinal diseases, especially Constipation.

 However, in epidemiological data, “Despite this, it is often poorly recognized, inadequately treated,” deserves better consideration. Functional Constipation is perfectly defined in the Rome II, III, and IV Criteria, and the treatments are not inadequate but face a series of problems linked to adherence.

 Relative to prevalence, In 2018, a systematic review and meta-analysis including 35 countries defined a pooled prevalence of 9.5%, with a similar prevalence in boys (8.6%) and girls (8.9%) but with a higher prevalence of 17.5% in children under four years (Koppen et al., 2018). Ref-1

 Concerning Overweight/Obesity, the potential association between FC and overweight and obesity, the statement of reduced average weight and height in children with chronic Constipation when compared to normal children is controversial, as there are a large number of studies favoring or denying this association. First, Koppen *et al*. (2016) included eight studies, and due to the heterogeneity of the study designs, they could not confirm the association between overweight/obesity and FC. ****However, other studies revealed the possible association between FC and excessive body weight. Second, a more recent systematic review and meta-analysis (Lazarus *et al*., 2022) ****evaluated eighteen studies involving 33,410 children and concluded that FC correlates with the prevalence of overweight and obesity. Ref-2, 3

 It was related to parents, home stressors, and bullying. In 2022 a systematic review included 15 articles (2954 children) with FC. It defined child maltreatment, divorce/separation of parents, severe illness in the family, and parental job loss as home stressors. In the same way, bullying, change in school, separation from the best friend, and failure on examination as school-related stressors were associated with childhood constipation (Liyanarachchi et al., 2022). 

 A recent good-quality systematic review of eleven studies evaluated the relationship between psychological stress and pediatric Functional Constipation. Similarly to the previous review, the authors classify psychological stressors into family, school, and related psychological disorders. In conclusion, most studies showed a significant relationship with Constipation, suggesting a collaborative effort between parents, children, and healthcare professionals (Gozali et al., 2023). Ref-4, 5

**Methods**

 The six stages of Arksey and O’Malley Framework are described in detail, as well as the Inclusion and exclusion criteria in Table 1.

Is the study design appropriate for the research question?

Yes

Is the rationale for, and objectives of, the study clearly described?

Yes

Are sufficient details of the methods provided to allow replication by others?

Yes

Are the datasets clearly presented in a useable and accessible format?

Yes

Reviewer Expertise:

Pediatric Gastroenterology (constipation, Quality of Life)

I confirm that I have read this submission and believe that I have an appropriate level of expertise to confirm that it is of an acceptable scientific standard.

## References

[1] : Prevalence of Functional Defecation Disorders in Children: A Systematic Review and Meta-Analysis. *J Pediatr* .2018;198: 10.1016/j.jpeds.2018.02.029 121-130.e6 10.1016/j.jpeds.2018.02.029 29656863

[2] : Functional Defecation Disorders and Excessive Body Weight: A Systematic Review. *Pediatrics* .2016;138(3) : 10.1542/peds.2016-1417 10.1542/peds.2016-1417 27531145

[3] : Relationship of Functional Constipation and Growth Status: A Systematic Review and Meta-Analysis. *J Pediatr Gastroenterol Nutr* .2022;75(6) : 10.1097/MPG.0000000000003600 702-708 10.1097/MPG.0000000000003600 36053122

[4] : Association between childhood constipation and exposure to stressful life events: a systematic review. *Neurogastroenterol Motil* .2022;34(4) : 10.1111/nmo.14231 e14231 10.1111/nmo.14231 34415089

[5] : Relationship between psychological stress with functional constipation in children: a systematic review. *Pan Afr Med J* .2023;46: 10.11604/pamj.2023.46.8.41130 8 10.11604/pamj.2023.46.8.41130 37928217 PMC10620441

# Response

**Published:** 2024-05-29

**Authors:** Yvonne McCague

**Affiliations:** SNMHS, University College Dublin, Dublin, Leinster, Ireland

The authors are very grateful for your time and effort in agreeing to review our scoping review protocol. We really appreciate your valuable suggestions and feedback with the relevant references as provided, and have made the relevant amendments and additions accordingly. Our itemised responses to your constructive feedback is detailed below: 

 1. The term "treated"  was amended to "managed" and problems associated with management was expanded upon as recommended (pg. 3).

 2. Global pooled prevalence added as recommended (pg. 4)

 3. The association of childhood FC and obesity was added as recommended. This factor was discussed in my methodology chapter as a justification for research from an Irish perspective, in view of recent publications regarding increasing obesity and overweight in children and adolescent in Ireland. (pg. 5)

 4. The association between stressful life events was expanded upon as recommended. This was a finding in my completed scoping review (pg. 7).

# Reviewer report: Reviewer response for version 1

**DOI:** 10.21956/hrbopenres.14999.r37448

**Published:** 2024-05-25

**Authors:** Glicia Estevam de Abreu

**Affiliations:** 1 Center for Children's Urinary Disorders (CEDIMI), Bahiana School of Medicine and Public Health,, Bahia, Brazil

This study is very interesting, given that it describes a protocol for research into childhood constipation and its psychosocial implications.

 The introduction provides a broad overview of childhood constipation, demonstrating the importance of the topic. However, I believe it is critical to include information about the link between constipation and urinary symptoms. This association, as well as its implications for the child's psychosocial development, has received a lot of attention.

 Although the description of the objectives is clear, I recommend mentioning how the articles defined constipation. Although the Rome IV Criteria are the most commonly used diagnostic tool for constipation, studies that do not use this tool are not uncommon. This could lead to misunderstandings.

 Methods: Nothing to add.

Is the study design appropriate for the research question?

Yes

Is the rationale for, and objectives of, the study clearly described?

Yes

Are sufficient details of the methods provided to allow replication by others?

Yes

Are the datasets clearly presented in a useable and accessible format?

Yes

Reviewer Expertise:

Constipation, Bladder and Bowel dysfunction

I confirm that I have read this submission and believe that I have an appropriate level of expertise to confirm that it is of an acceptable scientific standard.

# Response

**Published:** 2024-05-29

**Authors:** Yvonne McCague

**Affiliations:** SNMHS, University College Dublin, Dublin, Leinster, Ireland

The authors are very grateful for your time and effort in agreeing to review our scoping review protocol. We really appreciate your valuable suggestions and feedback, and have made the relevant amendments and additions accordingly. Our itemised responses to your constructive feedback is detailed below: 

 1. A discussion on the link between constipation and urinary symptoms expanded upon as recommended (pg. 5). 

 2. A description of ROME IV diagnostic took added as recommended (pg. 3).

# Reviewer report: Reviewer response for version 1

**DOI:** 10.21956/hrbopenres.14999.r36476

**Published:** 2023-10-31

**Authors:** Carol Joinson

**Affiliations:** 1 Centre for Academic Child Health, Population Health Sciences, Bristol Medical School, University of Bristol, Bristol, England, UK

The article describes a study protocol for a scoping review which is aimed at answering the research question, “What has been reported about the psychosocial implication of childhood constipation among children and their families?”.

 This is a well written protocol and the review is in an important and under-researched area of child health.  I have some recommendations for the authors:  

 Abstract:

 Please change “can be caused by” to “is associated with” in the following sentence: 

 “Primary or functional constipation (FC) has no known underlying pathology but can be caused by lifestyle, psychological, and behavioural factors.”

 Introduction:  

 I suggest moving paragraph 3 (which describes the prevalence of constipation) to the second paragraph.  It would be helpful for readers to know the prevalence before describing the research on possible risk factors and consequences.

 Is it possible to add prevalence estimates in community samples by age group? Does the prevalence of childhood constipation increase or decrease with age, or remain constant?

 Paragraph 2: there is epidemiological research (e.g. in the ALSPAC cohort) that is missing on early childhood psychosocial risk factors for constipation (e.g. stressful life events, child behaviour/emotional problems, exposure to maternal psychopathology).  This research is relevant to the content of this paragraph.

 Paragraph 4 describes associations between constipation, and reduced height and weight, in addition to higher somatic scores. It also describes a study by Appak et al (2017) that compared depression, anxiety and emotional/behaviour problems in children with constipation and healthy controls. The same study is mentioned again in paragraph 5 which begins: “Chronic constipation also has a significant psychosocial impact on children and adolescents”.  I suggest mentioning these findings relating to depression, anxiety and emotional/behaviour problems in paragraph 5. 

 A couple of limitations of the Appak et al. study are mentioned: small sample sizes and non-randomization of sampling. Please describe the limitations of the other studies e.g. study design, sample size, risk of bias. Did the studies adjust for confounders?  If so, what were they?

 Some studies (e.g. Wang et al. 2013) are described as a “cross-sectional, case-control study”. Please explain this study design.

 Rajindrajith et al.’s (2016) survey found that faecal incontinence associated with chronic constipation increased the likelihood of emotional abuse in school by other children and teachers.  Did this study adjust for potential confounders that might provide an alternative explanation for the observed association?

 The cost implications of constipation for families and the burden on healthcare services could be expanded. The review could also mention the importance of early identification and intervention for paediatric constipation to prevent it from becoming chronic and having secondary impacts on the child and family.

 In Aims and Objectives it would be helpful to define the term ‘psychosocial’ and to specify the domains that will be explored.

 Methods

 Please could you change the review question to “What are the psychosocial impacts of childhood constipation on children and their families?”

 Stage 6: Consultation Exercise and Dissemination: A useful addition to your stakeholders would be charities involved in supporting children and young people affected by chronic constipation and continence issues, and their families. Health professionals may not have insights into the full range of psychosocial impacts of constipation.

Is the study design appropriate for the research question?

Yes

Is the rationale for, and objectives of, the study clearly described?

Yes

Are sufficient details of the methods provided to allow replication by others?

Yes

Are the datasets clearly presented in a useable and accessible format?

Not applicable

Reviewer Expertise:

Paediatric incontinence; developmental psychology; epidemiology; mental health

I confirm that I have read this submission and believe that I have an appropriate level of expertise to confirm that it is of an acceptable scientific standard, however I have significant reservations, as outlined above.

# Response

**Published:** 2024-05-29

**Authors:** Yvonne McCague

**Affiliations:** SNMHS, University College Dublin, Dublin, Leinster, Ireland

The authors are very grateful for your time and effort in agreeing to review our scoping review protocol. We really appreciate your valuable suggestions and feedback, and have made the relevant amendments and additions accordingly. Our itemised responses to your constructive feedback is detailed below: 

 1. Amended as recommended from "can be caused by" to "is associated with" (pg. 3)

 2. Paragraph 3 moved to paragraph 2 (pg. 4). Prevalence estimates by age group and if influenced by age added (pg. 4). 

 3. Epidemiological study in the ASLPAC cohort by Joinson *et al *(2019) were added as recommended as this was very relevant to the content of this paragraph. (pg. 4)

 4. Findings relating to depression, anxiety and emotional/behaviour problems were added to the discussion regarding the significant psychosocial impact on children and adolescent as recommended (pg. 4)

 5. Limitations such as study design, sample size, risk of bias and confounding variables reported in other studies as recommended (pg. 4,5,6)

 6. Lack of adjustment for confounders reported as suggested (pg. 6)

 7. Discussion on cost factors and importance of early identification and intervention for paediatric constipation to prevent it from becoming chronic and having secondary impacts on the child and family expanded upon as recommended (pg. 6,7)

 8. The term psychosocial and domains to be explored expanded upon as recommended (pg. 11)

 9. Question amended as recommended (pg. 9)

 10. Additional stakeholders such as charities involved in supporting children and young people affected by chronic constipation and continence issues, and their families expanded upon as recommended (pg. 14)
